# Identification of Suppressor of Clathrin Deficiency-1 (*SCD1*) and Its Connection to Clathrin-Mediated Endocytosis in *Saccharomyces cerevisiae*

**DOI:** 10.1534/g3.118.200782

**Published:** 2019-01-24

**Authors:** Balaji T. Moorthy, Anupam Sharma, Douglas R. Boettner, Thomas E. Wilson, Sandra K. Lemmon

**Affiliations:** * Department of Molecular and Cellular Pharmacology, University of Miami, Miller School of Medicine, Miami, FL,; † Department of Pathology, University of Michigan Medical School, Ann Arbor, MI,; ‡ Department of Human Genetics, University of Michigan Medical School, Ann Arbor, MI

**Keywords:** Clathrin, endocytosis, membrane trafficking, pooled linkage analysis

## Abstract

Clathrin is a major coat protein involved in vesicle formation during endocytosis and transport in the endosomal/trans Golgi system. Clathrin is required for normal growth of yeast *(Saccharomyces cerevisiae)* and in some genetic backgrounds deletion of the clathrin heavy chain gene (*CHC1*) is lethal. Our lab defined a locus referred to as “*s*uppressor of *c*lathrin *d*eficiency” (*SCD1*). In the presence of the *scd1-v* allele (“v” – viable), yeast cells lacking clathrin heavy chain survive but grow slowly, are morphologically abnormal and have many membrane trafficking defects. In the presence of *scd1-i* (“i”- inviable), *chc1∆* causes lethality. As a strategy to identify *SCD1*, we used pooled linkage analysis and whole genome sequencing. Here, we report that *PAL2* (*YHR097C*) is the *SCD1* locus. *pal2∆* is synthetic lethal with *chc1∆*; whereas a deletion of its paralog, *PAL1*, is not synthetic lethal with clathrin deficiency. Like Pal1, Pal2 has two NPF motifs that are potential binding sites for EH domain proteins such as the early endocytic factor Ede1, and Pal2 associates with Ede1. Also, GFP-tagged Pal2p localizes to cortical patches containing other immobile phase endocytic coat factors. Overall, our data show that *PAL2* is the *SCD1* locus and the Pal2 protein has characteristics of an early factor involved in clathrin-mediated endocytosis.

Movement of proteins within the secretory and endocytic pathways is initiated by the binding of coat proteins to the cytosolic surface of the membrane followed by capture of cargo molecules and vesicular budding (McMahon and Mills 2004, Gomez-Navarro and Miller 2016). Clathrin and its associated proteins form a major class of vesicular transport coats (Kirchhausen *et al.* 2014, Robinson 2015). Clathrin-coated vesicles (CCVs) are involved in receptor mediated endocytosis, recycling of membranes, transcellular transport and transport between the TGN and endosomes (Kirchhausen *et al.* 1989, Pearse and Robinson 1990, Bonifacino and Glick 2004, Boettner *et al.* 2011, Kirchhausen *et al.* 2014, Robinson 2015, Elkin *et al.* 2016, Lu *et al.* 2016). Clathrin, which forms the striking polygonal surface lattice on CCVs, is a trimeric molecule, or triskelion, containing three heavy chains (HC) of ∼180 kD that radiate from a vertex and three light chains (LC) of 30 - 40 kD, which bind noncovalently near the vertex of the triskelion, one per heavy chain arm (Kirchhausen *et al.* 1989, Edeling *et al.* 2006).

CCVs have been found in virtually every eukaryotic organism that has been examined, including the yeast *Saccharomyces cerevisiae* (Mueller and Branton 1984, Payne and Schekman 1985, Lemmon and Jones 1987, Silveira *et al.* 1990). Previous studies in yeast found that deletion of the clathrin HC gene, *CHC1*, is lethal in some genetic backgrounds, but not in others (Payne and Schekman 1985, Lemmon and Jones 1987, Payne *et al.* 1987, Schekman and Payne 1988, Munn *et al.* 1991). The viable clathrin HC deficient cells exhibit a number of phenotypes, including slow growth, abnormal morphology and polyploidy, and defects in mating, sporulation, endocytosis and sorting in the endosomal/Trans Golgi Network (TGN) system (Payne and Schekman 1985, Lemmon and Jones 1987, Payne *et al.* 1987, Payne *et al.* 1988, Payne and Schekman 1989, Lemmon *et al.* 1990, Nelson and Lemmon 1993, Kaksonen *et al.* 2005, Newpher and Lemmon 2006). In strains studied in this laboratory, we uncovered a polymorphism in an independently segregating genetic locus that causes lethality in *chc1∆* cells (Lemmon and Jones 1987). We called this locus “*s*uppressor of *c*lathrin *d*eficiency” (*SCD1*), where in the presence of the *scd1-v* allele, yeast cells lacking clathrin HCs are **v**iable but with the *scd1-i* allele clathrin HC deficient cells are **i**nviable. However, the genetic basis for the lethality in *scd1-i chc1∆* cells has remained a mystery for over 30 years. We initially sought to identify the *SCD1* gene using a multicopy suppressor screen, and identified the genes *SCD2 – SCD6* whose overexpression could supress the lethality of clathrin HC-deficient yeast cells carrying the *scd1-i* allele (Nelson and Lemmon 1993). However, segregation analysis showed that none of these genes were allelic to the *SCD1* locus ((Nelson and Lemmon 1993, Gelperin *et al.* 1995, Nelson *et al.* 1996, Huang *et al.* 1997), Gelperin and S. Lemmon unpublished observations). Also, traditional complementation and other chromosomal mapping methods to identify the gene were complicated by the fact that *chc1∆* cells become polyploid at high frequency and are difficult to transform (Lemmon and Jones 1987, Lemmon *et al.* 1990).

More recently pooled linkage analysis and whole genome sequencing has been used in yeast to identify difficult to clone mutations, polymorphisms or dominant alleles (Shendure and Ji 2008, Birkeland *et al.* 2010, Song *et al.* 2014, Lang *et al.* 2015, Linder *et al.* 2016). Taking advantage of this approach we now report that *scd1-i* encodes a mutation in the *PAL2* (*YHR097C*) locus. *pal2∆* is synthetic lethal with *chc1∆*, whereas a knockout of its paralog, *PAL1*, an endocytic factor (Carroll *et al.* 2012), is not synthetic lethal with clathrin heavy chain deficiency. Pal2-GFP localizes to cortical patches, similar to Pal1-GFP and other endocytic coat factors. Overall, our data show that *PAL2* is the *SCD1* locus and is likely involved in clathrin-mediated endocytosis.

## Materials and Methods

### Yeast strains and methods

*S. cerevisiae* strains used in this study are related to S288c and are listed in Tables S1 and S2. Standard methods were employed for DNA manipulations, and yeast tetrad analysis, growth and transformation. Unless otherwise indicated, the Longtine method was used for generating the fluorescently tagged reporters and deletion mutants (Longtine *et al.* 1998). For the triple tagged Pal1-3xGFP, *pFA6a 3xGFP-KanMX6* was used as a template (Kovar *et al.* 2005).

For growth tests shown in Figure 2 and Figure S2 strains were streaked from a single colony onto 1% yeast extract + 2% peptone (YEP) containing 2% galactose (YEP-Gal) and YEP + 2% Dextrose (YEPD) plates and incubated at 30° (Figure 2), 34° and 37° (Figure S2) for 4 days. For serial dilution spotting in Figure 2B, the strains were grown overnight in YEP liquid medium containing 2% galactose (YEP-Gal). Cells from these cultures were inoculated into fresh medium at 0.025 × 10^7^ cells/ml and then grown to ∼1.0 × 10^7^ cells/ml. Cells were pelleted, washed with dH_2_O and resuspended in YEPD at 0.1 × 10^7^ cells/ml, and allowed to grow for ∼14 hr. Serial ten-fold dilutions were made in dH_2_O. Five µl of each of the 10^−1^ to 10^−4^ dilutions were spotted on YEP-Gal and YEPD plates and grown for 4 days.

### Plasmids

Plasmids used in this study are listed in Table S3. *pRS426-PAL2* was generated by PCR amplification of *PAL2 (YHR097C)* with ∼500 base pairs (bp) upstream and ∼300 bp downstream of the open reading frame (ORF) using clone no. 671 (plate no. 7; F12 well) from the multicopy Tiling library collection (gift of G. Prelich (Jones *et al.* 2008)) as a template. The PCR product was sequenced to confirm it encoded the wild type Pal2 protein (*scd1-v* background). The amplified PCR fragment was digested with *Kpn*1 (5′) and *Bam*H1 (3′), which had been encoded in the primers, and cloned into the pRS426 vector cut with *Kpn*1 and *Bam*H1. Plasmid *pRS316-PAL2* was generated in the similar way as pRS426-*PAL2* except that both the vector and PCR fragment were digested with *Xho*1 (5′) and *Kpn*1 (3′). Plasmid clones were verified by restriction digestion and sequencing.

Plasmid pET28c*-EDE1 (EH1-3)* [pBW1161] (gift of B. Wendland), contains 1261 bp of the coding sequence of the N-terminal region of *EDE1* for EH domains 1, 2 and 3 inserted into the *Bam*H1 (5′) and *Xho*1 (3′) sites of *pET28c*. This was used for bacterial expression of a N-terminal His6-tagged EH1-3 domain. The coding sequence of GFP was inserted between the *Nde*1 (5′) and *Not*1 (3′) sites of *pET22b*, to generate pET22b*-GFP* to express His6-GFP (gift of D. Patel & F. Zhang). Plasmids were verified by restriction digestion and sequencing.

### Whole genome sequencing

The strains used in this analysis were generated from parents and spore segregants previously described in (Lemmon and Jones 1987) (see Table S1 for the list of strains used for *SCD1* identification). Parent strains BJ2700 (*CHC1leu2scd1-i*) and BJ2738 **(***CHC1leu2scd1-v*) were crossed to generate the diploid BJ3068 (*CHC1/CHC1leu2/leu2scd1-i/scd1-v*). Then *CHC1* was disrupted with *LEU2* to generate *chc1∆:LEU2/CHC1scd1-i/scd1-v* transformants BJ3119 and BJ3120. These were subjected to tetrad analysis and wild type *CHC1scd1-i* or *scd1-v* spores were identified based on their segregation from tetrads with four viable spores or 2 viable spores, respectively. The parents of BJ3068 (BJ2700 and BJ2738) and 10 each of *CHC1scd1-i* or *CHC1scd1-v* spore segregants were analyzed.

Individual cultures of 10 segregants bearing the *scd1-v* allele or 10 bearing the *scd1-i* allele were grown in YEPD. Equal numbers of cells (1x10^7^) from each pool member were mixed and genomic DNA extracted using the YeaStar Genomic DNA Kit (D2002; Zymo Research, Irvine CA). Cultures of parents were treated similarly except 1x10^8^ cells were harvested for DNA isolation.

IIllumina sequencing of parents and pooled strains and alignment of the resulting reads were performed as described previously (Birkeland *et al.* 2010, Yau *et al.* 2014) by the Center for Genome Technology Sequencing Core at the Hussman Institute for Human Genomics, University of Miami, Miller School of Medicine. All samples were prepped via ’TruSeq DNA SamplePrep Guide 15026486 C’ (Illumina) with an input of 700-800ng DNA and 12 cycles of PCR. Five hundred bp long libraries were created and the two pools and parent samples were sequenced in a single multiplexed HiSeq lane with 2x101 nt paired end reads. Read alignment to the sacCer3 (R64.1.1) (Engel *et al.* 2014) version of the yeast genome was performed using BWA-MEM using default parameters (Li and Durbin 2010). Because of the much higher read depth in the current study, a novel approach to data analysis used samtools (samtools mpileup -DSu -d 1000 -L 1000) followed by bcftools (bcftools view -bvcg -T pair, with a ploidy of 2) to identify variants for which there was a high likelihood that the composite genotype was different for the *scd1-i* and *scd1-v* pools. Randomly segregating alleles would each appear as heterozygous in each pool while those linked to the causative mutant allele would appear as either homozygous reference or variant in different pools. The same analysis with a ploidy of 1 compared the two starting haploid strains, where candidate alleles must again have a high likelihood of having a different genotype. The two outputs were filtered for variants with a Phred-scaled log ratio of genotype likelihoods (CLR score) >225 in each of the pool and haploid strain comparisons, which resulted in two variants that proved to have only a 2 bp separation on chrVIII. Read counts (forward/reverse strand, sacCer3 coordinates) for chrVIII:298485, T > C were: wild-type pool (*scd1-v*), 157/90 reference and 15/12 mutant; mutant pool (*scd1-i*), 0/0 reference and 206/113 mutant. Read counts for chrVIII:298487, A > C were: wild-type pool (*scd1-v*), 157/90 reference and 20/14 mutant; mutant pool (*scd1-i*), 0/0 reference and 237/115 mutant. The most likely explanation for the presence of rare mutant reads in the wild-type pool was imperfect scoring of contributing spore clones.

### Microscopy and image analysis

For most experiments, cells were grown to log phase at 30° in synthetic medium, concentrated, and immobilized on Concavalin-A coated coverslips. Coverslips were then mounted on slides, and imaged at 25° as indicated below.

Localization of Pal2-GFP or Pal1-(3x) GFP, and co-localization with other markers was performed on an Olympus fluorescence BX61 upright microscope equipped with Nomarski differential interference contrast (DIC) optics, a Uplan Apo 100x objective (1.35 NA), a Roper CoolSnap HQ camera, and Sutter Lambda 10-2 excitation and emission filter wheels, and a 175 watt Xenon remote source with liquid light guide. Image capture was automated using Intelligent Imaging Innovations Slidebook 6 for Windows 7. Z-stacks of 0.25 μm of fields of cells were taken and a medial plane was selected for image analysis. Image analysis was carried out using Slidebook 6 software and later exported to TIF files. The images of 300 dpi were then cropped and arranged in Adobe Photoshop CS5 and Creative Cloud. Approximately 40 – 60 cells were considered for localization of Pal2-GFP and Pal1-3xGFP. For the quantification of Pal2-GFP patches containing Ede1-mCherry, Sla2-RFP, End3-mCherry and Abp1-RFP, 10 – 18 cells with distinct Pal2-GFP patches from a single medial plane were selected and examined for the presence/overlap of mCherry/RFP signal. The percentage of Pal2-GFP patches containing Ede1-mCherry, Sla2-RFP, End3-mCherry and Abp1-RFP was calculated by the ratio between the number of GFP/RFP(mCherry) overlapping patches to the total number of Pal2-GFP patches in the cells. The percentage overlap of Pal1-3xGFP patches with Pal2-mCherry patches was determined in a similar way. Statistics were performed using the GraphPad Prism 7 software. Two-tailed *t*-test was carried out for each pair to measure the significance of the data.

Cortical patch to cytosol fluorescence intensity ratio analysis was carried out on the Olympus fluorescence BX61 upright fluorescence microscope as described previously (Chi *et al.* 2012) using Slidebook 6.0 for Windows 7 for acquisition and analysis. Strains were grown at 30° to early log phase and z-stack images (5 × 0.25 um) were captured. Analysis was performed on a medial focal plane. The fluorescence intensity of the brightest cortical patch in a cell was divided by the fluorescence from the mother cell cytosol. A representative background intensity value (outside the cell) was also subtracted from both patch and mother cell cytosol intensities before calculating the patch/cytosol ratio (n ≥ 25 cells for each strain).

To prevent actin polymerization and inhibit internalization at endocytic sites, log phase cells were treated in synthetic medium supplemented with 200 μM Latrunculin-A (LAT-A; Enzo, BML-T119) for 1 h at 30°. Control cells were treated in medium containing an equal volume of dimethyl sulfoxide (DMSO), the diluent for LAT-A.

### Protein purification and pull-down experiment

His_6_-Ede1 (EH1-3) expressed from pET28c*-EDE1(EH1-3)* or His_6_-GFP expressed from pET22c-GFP were purified on Ni-NTA agarose beads (Cat No./ID: 30230, Qiagen) according to the manufacturer’s instructions. Yeast extracts were made from 8x10^8^ cells grown in YEPD to log phase. Cell lysates were prepared by resuspending each cell pellet in 1ml of lysis buffer (10 mM Tris pH 8.0, 140 mM NaCl, 0.1% Tween-20, 1 mM β-mercaptoethanol, 1mM PMSF and 1x protease inhibitor cocktail {Protease Inhibitor Cocktail (100X); Cell Signaling, Catalog no.5871}) and subjecting it to glass bead vortexing (4 times – 1 min vortex and 2 min pause on ice), followed by centrifugation at 15000 × g in an eppendorf 5415R centrifuge for 10 min at 4°. An aliquot of supernatant was saved as input. For pull down experiments, 50 μl of Ni-NTA agarose slurry was equilibrated with lysis buffer and incubated with ∼30 μg of purified His_6_-Ede1 (EH1-3) or His_6_-GFP at 4° for 1.5 hr. Beads were washed three times with 1ml of wash buffer (lysis buffer + 20mM imidazole). Then 200μl of lysate was added to the bead alone or beads containing purified His_6_-Ede1(EH1-3) or His_6_-GFP, followed by incubation at 4° for 2 hr on a rotator. Beads were washed three times with wash buffer (lysis buffer without protease inhibitors + 10% glycerol) and resuspended in 30μl lysis buffer and 30μl of 4x SDS sample buffer. Samples were heated at 65° for 5 min and stored at -20° until used for immunoblot analysis. Twenty µl of samples from pull downs and 5μl of cell extracts for loading controls were run on Biorad precast 4–20% gradient mini gels and transferred to nitrocellulose membrane using Biorad Trans-blot turbo system (25V for 7 min). Pal2-GFP and Pal1-GFP were detected using anti-GFP antibody (1:1000, Roche; mouse monoclonal; catalog no. 11814460001), and His_6_-GFP and His_6_-Ede1 (EH1-3) were detected with 6x-His Tag monoclonal antibody (1:1000, Thermo Scientific; catalog no. 4A12E4). Goat anti-mouse conjugated with horseradish peroxidase (HRP) was used as the secondary antibody at 1:5000 dilution (Thermo Scientific; catalog no. 31430). The proteins were then detecting by Chemiluminescence using SuperSignal West Femto Maximum Sensitivity Substrate (Thermo Scientific; catalog no 34095).

### Data availability

Strains and plasmids are available upon request. Figure S1 shows the sequence alignment of the “Pal domain” of Pal2 and Pal1. Figure S2 shows the growth phenotype of *GAL1:CHC1* strains with the indicated genotypes streaked on YEP+glucose at elevated temperatures. Figure S3 shows the patch to cytosol fluorescence intensity ratio for Pal2-GFP and Pal1-3xGFP in wild type and different endocytic mutants. Figure S4 shows the patch to cytosol fluorescence intensity ratio for Sla1-GFP in wild type and *pal∆* mutants. Table S1 contains the yeast strains used for pooled linkage analysis and whole genome sequencing. Table S2 and Table S3 show the list of yeast strains and plasmids used in this study, respectively. Table S4 shows the tetrad data demonstrating that *pal1****∆*** is not synthetic lethal with *chc1****∆***. Supplemental material available at Figshare: https://doi.org/10.25387/g3.7582868.

## Results

### Identification of the SCD1 locus using pooled linkage analysis and next generation whole genome sequencing

**Figure 1:**
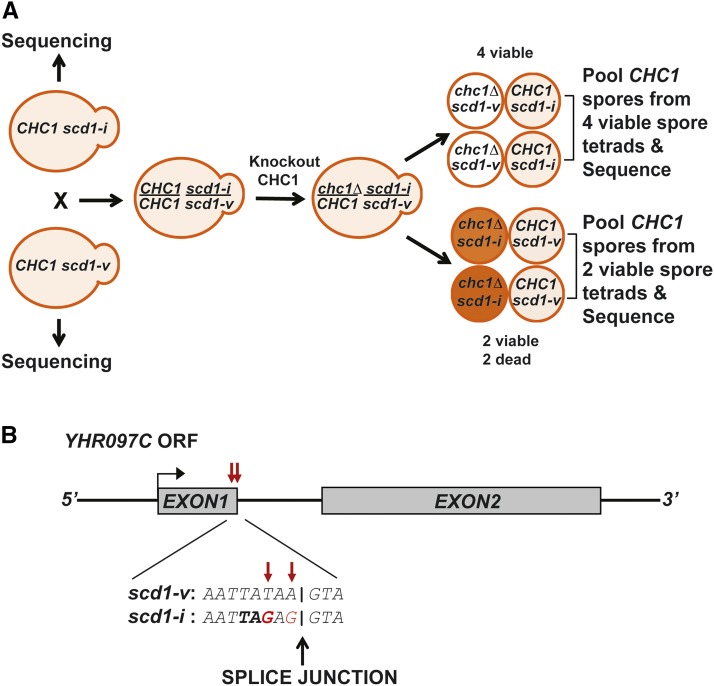
Identification of *SCD1* locus by next generation sequencing (NGS). (A) *CHC1* strains with the *scd1-v* (BJ2738) and *scd1-i* allele (BJ2700) were crossed to each other. *CHC1* was deleted from the resulting heterozygous (*scd1-i/scd1-v)* diploid (BJ3068). Tetrads were dissected and wild type spores were selected from tetrads with 4 viable spores (*CHC1 scd1-i*) or 2 viable spores (*CHC1 scd1-v*). Parents of the original diploid and pools of 10 *CHC1* spore segregants of *scd1-i* or *scd1-v* genotype were sequenced. (B) Schematic representation of *YHR097C*. Exon1 and Exon2 are shown as dark gray boxes. The red arrows indicate the mutation(s), where the first mutation leads to a Tyr residue to Stop codon at codon 41 of the *scd1-i* allele just upstream of the 5′ splice junction.

To discover functional mutations in yeast among a large excess of polymorphisms and incidental mutations, a strategy was developed based on next-generation sequencing, called pooled linkage analysis (Birkeland *et al.* 2010, Song *et al.* 2014, Lang *et al.* 2015, Linder *et al.* 2016). We employed the same strategy for the identification of the *SCD1* locus. In the original studies where this polymorphism was observed, two wild type strains had been crossed; one carrying the *scd1-v* allele (BJ2738) and the other bearing the *scd1-i* allele (BJ2700) generating a *scd1-v/scd1-i* heterozygous diploid (BJ3068) (Lemmon and Jones 1987) (see Table S1). The clathrin heavy chain gene (*CHC1*) was knocked out in the *scd1-v/scd1-i* heterozygous diploid and tetrads were dissected (Figure 1A). Tetrads from these crosses were saved enabling us to recover wild type spore segregants carrying the *scd1-i* or *scd1-v* allele (see Table S1, Figure 1A). Wild type spores of *scd1-i* genotype were selected from tetrads with four viable spores, since the *scd1-v* allele would have segregated with the two viable *chc1∆* spores. Wild type spores of *scd1-v* genotype were selected from tetrads with 2 viable spores, as the *scd1-i* allele would have segregated with the two dead *chc1-∆* spores. Parents of the original diploid cross and individual pools of 10 wild type spore segregants of *scd1-i* or *scd1-v* genotype were subjected to genomic DNA sequencing (Figure 1A). Incidental polymorphisms will segregate randomly and both alleles would be represented in the pools. However, causative *scd1* alleles that co-segregate with the clathrin heavy chain deficient cell phenotype will be homogenous in each pool. Only one candidate allele in the *scd1-i* pool and the original *scd1-i* parent (BJ2700) emerged from this analysis, which proved to be a pair of closely spaced single-nucleotide substitutions, chrVIII:298485,T > C and chrVIII:298487,A > C in *YHR097C*, a spliced gene (see Materials and Methods). Careful analysis of the sequencing results showed a mutation just upstream of the 5′ splice junction, where codon 41 for tyrosine is mutated to a stop codon (Figure 1B). *YHR097C* has a paralog *PAL1 (YDR348C)* that arose from a whole genome duplication (Byrne and Wolfe 2005). Intriguingly, Pal1 has been reported to be involved in clathrin-mediated endocytosis (Carroll *et al.* 2012). From sequence alignment, both proteins are 43% identical. Furthermore, both share 65% identity over a conserved “Pal domain” (Figure S1). We adopted the name *PAL2* for *SCD1* as Kaksonen and co-workers used this gene name in a prior study (Brach *et al.* 2014).

### pal2∆, but not pal1∆, is synthetically lethal with clathrin heavy chain deficiency

**Figure 2:**
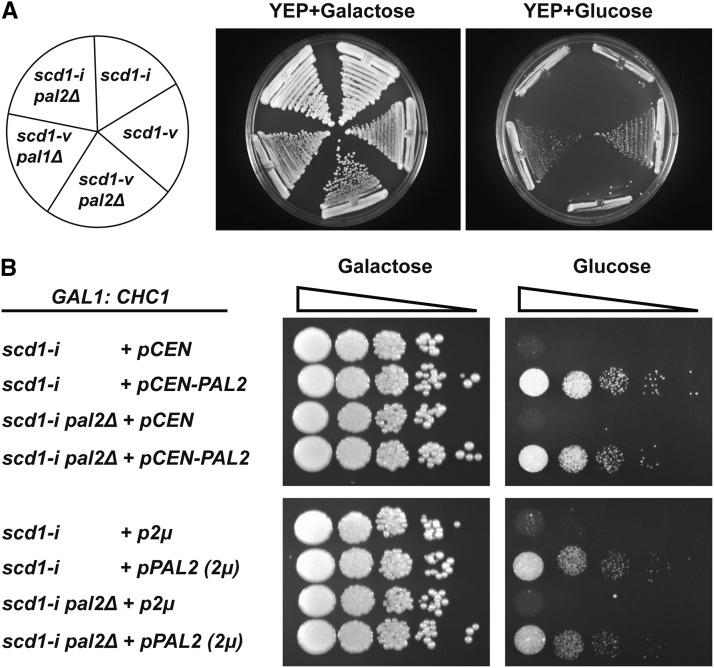
*pal2∆* is synthetic lethal with loss of clathrin heavy chain. (A) *GAL1:CHC1* strains with the indicated *SCD1* and *PAL1* genotypes were streaked on galactose and glucose medium and grown for 4 days. Strains used are: *scd1-i* (SL214); *scd1-v* (SL350); *scd1-v pal2∆* (SL7249); *scd1-v pal1∆* (SL7261); *scd1-i pal2*∆ (SL7251). (B) *GAL1:CHC1* strains with the indicated *SCD1* and *PAL1* genotypes were transformed with vector control (p*CEN* or p2μ) or plasmids expressing *PAL2* (p*CEN-PAL2* or p*PAL2* 2μ). The strains were grown to log phase in YEP-galactose and then transferred to YEP-glucose for ∼14 hr. Then 10-fold serial dilutions were spotted on galactose and glucose plates and grown for 4 days. Strains used are: *scd1-i +pCEN* (SL7444); *scd1-i +pCEN-PAL2* (SL7464); *scd1-i pal2∆ +pCEN* (SL7447); *scd1-i pal2∆ +pCEN-PAL2* (SL7448); *scd1-i +p2μ* (SL7278); *scd1-i +pPAL2 (2μ)* (SL7279); *scd1-i pal2∆ +p2μ* (SL7280); *scd1-i pal2∆ +pPAL2 (2μ)* (SL7281).

We previously showed that *CHC1* expressed via the *GAL1* promoter confers regulated control of clathrin HC synthesis (Nelson and Lemmon 1993). On galactose medium *GAL1:CHC1* cells express clathrin HC and grow normally, but on glucose medium *CHC1* expression is repressed and cells either grow slowly (*scd1-v*) or are inviable (*scd1-i*) (Figure 2A). We then deleted *PAL2* in these two strains (Figure 2A). The strains grew normally on galactose, but deletion of *PAL2* in the *GAL1:CHC1scd1-v* background now led to inviability on glucose, similar to the *GAL1:CHC1scd1-i* strain. In contrast, deletion of *PAL1* did not cause inviability nor did it further impair growth of the original *GAL:CHC1scd1-v* strain on galactose or glucose (Figure 2A), even at elevated temperature of 34° (Figure S2). All of the strains were inviable on glucose at 37°, as expected for cells lacking clathrin HC (Figure S2). These results indicate that *pal2∆*, but not *pal1∆*, is synthetic lethal with clathrin HC-deficiency.

We next tested whether *PAL2* could complement *GAL1:CHC1scd1-i* in glucose medium, and found that *PAL2* on a CEN plasmid indeed rescued the growth defect of *GAL1:CHC1scd1-i* strains (with or without *pal2∆*) (Figure 2B, upper panels). In an attempt to identify *SCD1* earlier, we performed a multi-copy suppressor screen on *GAL1:CHC1scd1-i* yeast. *SCD2-SCD6* were identified in this screen, but none were allelic to the *SCD1* locus ((Nelson and Lemmon 1993, Gelperin *et al.* 1995, Nelson *et al.* 1996, Huang *et al.* 1997), unpublished observations). We might have expected to identify *PAL2* in this screen, so we considered the possibility that overexpression of *PAL2* might cause impaired growth of wild type yeast or Chc^-^ cells. But overexpression of *PAL2* (p*PAL2* (2µ)) also rescued *GAL1:CHC1scd1-i* strains (with or without *pal2∆*) on glucose and had no effect on cell growth when *CHC1* was expressed on galactose medium (Figure 2B, lower panels).

### YHR097C (PAL2) is SCD1 locus

Our pooled linkage/sequence analysis indicates that *PAL2* is *SCD1*. Supporting this we so far have shown that *pal2∆* causes synthetic lethality in clathrin HC deficient yeast and *PAL2* complements the *scd1-i* mutation. However to prove that *PAL2* is the *SCD1* locus, a classical genetic approach is required. To this end, we first deleted *PAL2* in *CHC1scd1-v* cells and crossed this (*CHC1pal2∆:NATMx6*; SL7098) with a *chc1∆:LEU2scd1-i* strain where the spore segregant came out of tetrads protected by YCp50-*CHC1* (SL98). The plasmid was dropped from the *chc1∆/CHC1* heterozygous diploid and subjected to tetrad analysis. If *PAL2* is *SCD1*, then the diploid strain would be homozygous at the *SCD1 locus (pal2∆:NatMx6/scd1-i)* and we expect 2 viable *CHC1* and 2 dead spores (*chc1∆:LEU2* with either *scd1-i* or *pal2∆:NATMx6*) in all tetrads. If *PAL2* is not the *SCD1* locus, then some of the *chc1∆* spores would survive by receiving the *scd1-v* allele from the *CHC1scd1-v* parent. Twenty dissected tetrads segregated 2 viable:2 dead with all viable spores being *CHC1* Leu^-^ (Table 1). When we performed the same experiment in the presence of YCp*CHC1* (*URA3*) the plasmid rescued the growth of *chc1∆:LEU2* spores (Leu^+^Ura^+^), but we did not recover any Chc^-^ spores growing in the absence of the plasmid (Leu^+^Ura^-^) (Table 1). This confirms that *PAL2/YHR097C* is the *SCD1* locus, and *scd1-i* is a truncation mutant of *PAL2*.

**Table 1:** pal2∆ is synthetic lethal with chc1∆

Diploid genotype SL98 X SL7098	No. of tetrads with ratio of viable to dead spores			No. of spores with phenotype			
	4:0	3:1	2:2	Leu^-^Ura^±^	Leu^+^Ura^+^	Leu^+^Ura^-^	Dead
*chc1∆:LEU2/CHC1 scd1-i/pal2∆:NatMX6*	0	0	20	40	0	0	40
*chc1∆:LEU2/CHC1 scd1-i/pal2∆:NatMX6 (*YCp50*-CHC1)*	11	10	11	58	38	0	32

Strains SL98 and SL7098 were crossed. The heterozygous diploid was then sporulated (in the absence and presence of plasmid YCp50*-CHC1*), followed by tetrad dissection. Data in the table represent the number of tetrads with ratio of viable to dead spores and number of spores with different phenotypes. If *pal2∆* is integrated at the *SCD1* locus, we expect no viable Leu^+^ Ura^-^ (*chc1∆)* spores in the absence of the *CHC1*, *URA3* plasmid, YCp50-*CHC1*.

We also tested whether *pal1∆* is synthetic lethal with *chc1∆*, but as shown in the *GAL1:CHC1* shut down experiments (Figure 2A), *pal1∆* does not cause inviability with clathrin HC deficiency. This was confirmed in tetrads from crosses of *pal1∆ scd1-v* and *chc1∆* YCp*CHC1scd1-v*, where viable *chc1∆:LEU2pal1∆:KanMX6* spores were recovered (Table S4*)*.

### Localization of Pal2-GFP and Pal1-GFP

**Figure 3:**
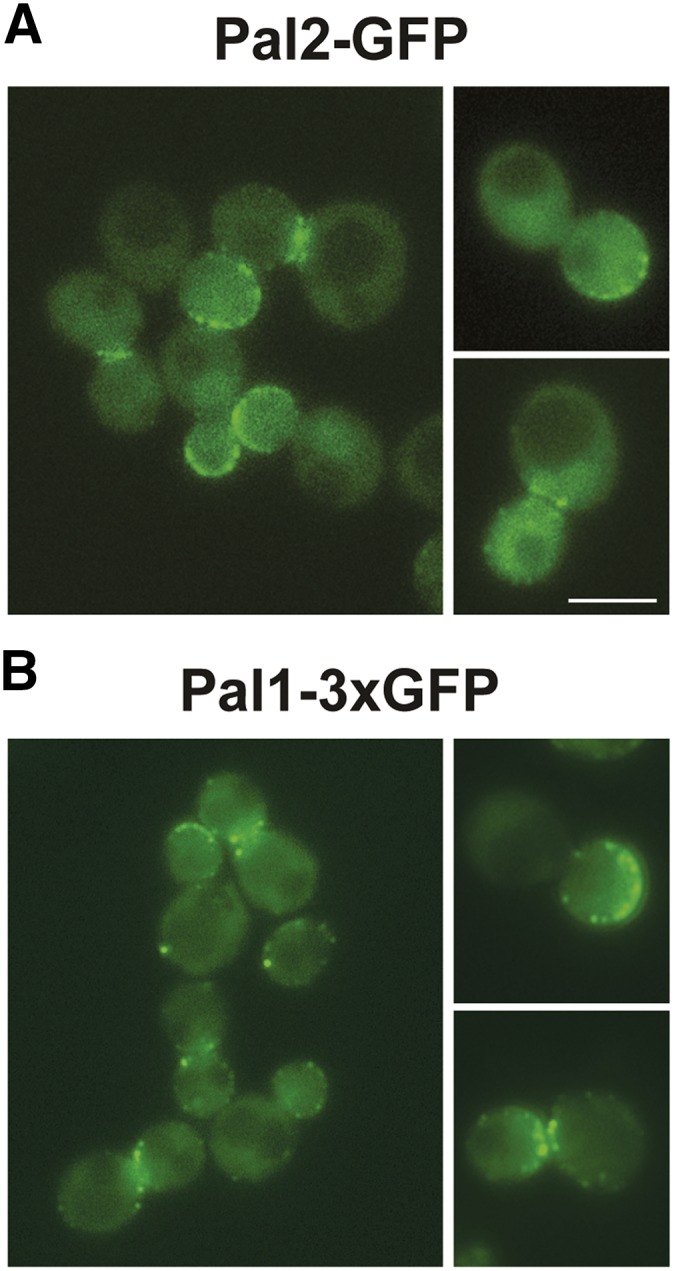
Pal2 and Pal1 localize to to cortical patches. (A) Pal2-GFP (SL7455); (B) Pal1-3xGFP (SL7335) were imaged by fluorescence microscopy. Shown are images from a single medial plane of a z-stack. Scale bar: 5μm.

The clathrin endocytic pathway in yeast initiates with an immobile phase where the early endocytic factors, such as Syp1, Ede1 and clathrin, are first recruited to cortical patches, followed by the assembly of mid-late coat factors (*e.g.*, Sla2, Sla1, Ent1/2). Ede1 and Syp1 leave the cortex just as the rapid mobile actin driven invagination phase commences, which is followed by scission of the coated vesicle. After the release of the nascent vesicle, it is rapidly uncoated and moves inwards and coat factors are recycled for the next round of endocytosis (Boettner *et al.* 2011, Weinberg and Drubin 2012, Goode *et al.* 2015). Previous studies have shown that Pal1-GFP forms patches at the cell cortex that are characteristic of endocytic coat proteins and it is considered an early arriving endocytic factor (Carroll *et al.* 2012). Global analysis of protein localization reported that GFP-tagged Pal2 localizes to the cytoplasm and nucleus (Huh *et al.* 2003). However, due to the homology with Pal1, we speculated that Pal2 might also localize to endocytic sites. We generated a strain expressing Pal2 with a C-terminal GFP tag. GFP-tagged Pal2 is functional as we obtained viable spores in genetic crosses with *chc1∆* (not shown). Since Pal1-GFP was difficult to visualize in prior studies (Carroll *et al.* 2012), we generated a strain that expresses Pal1 with a C-terminal 3xGFP tag. However, *pal1∆* has no phenotype even in combination with *chc1∆* or *pal2∆* (see below), so it was not possible to confirm Pal1-3xGFP function. But it behaved much like Pal1-GFP studied previously (Carroll *et al.* 2012). When we visualized the Pal proteins in live cells, we found more than 76% of cells (n = 60) had cortical patch localization (Figure 3A, 3B), a characteristic of endocytic coat proteins. Also, in addition to the cell cortex, Pal2-GFP was observed at the bud neck (32% of cells; n = 60), similar to Pal1-3xGFP (38% of cells; n = 60; Figure 3A, 3B).

**Figure 4:**
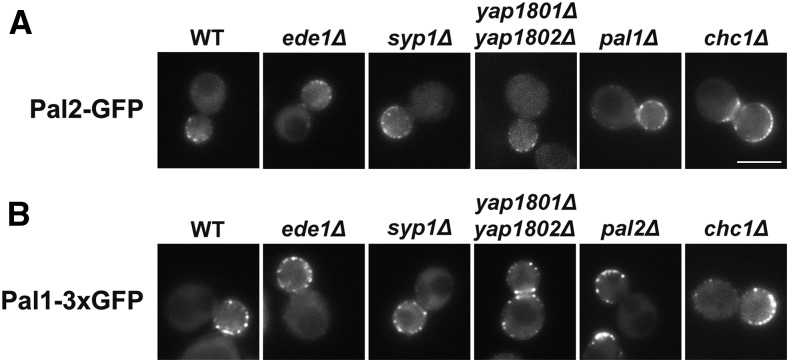
Localization of Pal2-GFP and Pal1-3xGFP in the absence of other endocytic factors. (A) Yeast strains expressing Pal2-GFP (WT; SL7455) and in *ede1∆* (SL7505), *syp1∆* (SL7405), *yap1801/2∆* (SL7401), *pal1∆* (SL7475), *chc1∆* (SL7451). (B) Yeast strains expressing Pal1-3xGFP (WT; SL7335) and in *ede1∆* (SL7502), *syp1∆* (SL7579), *yap1801/2∆* (SL7581), *pal2∆* (SL7400), *chc1∆* (SL7468). Images shown are from a single plane of a z-stack focused on the middle of the cell. Scale bar: 5μm.

Since *pal2∆* shows synthetic lethality with *chc1∆* and its paralog Pal1 is an early endocytic factor, we next tested growth phenotypes of *pal2∆* with deletions of *PAL1* and other early endocytic factor genes, *EDE1*, *SYP1* and *YAP1801/2* at normal (30°) and elevated temperatures (34° and 37°), but saw no effect either in tetrads or by direct gene deletions (data not shown). We then examined whether early endocytic proteins, Ede1, Syp1, Yap1801/2, and clathrin HC (Kaksonen *et al.* 2005, Newpher *et al.* 2005, Toshima *et al.* 2006, Boettner *et al.* 2009, Carroll *et al.* 2009, Reider *et al.* 2009, Stimpson *et al.* 2009, Carroll *et al.* 2012), are required for the recruitment of Pal2-GFP or Pal1-3xGFP to cortical sites. However, both Pal2-GFP and Pal1-3xGFP localization to cortical patches and the bud neck in null mutants of each of these genes was similar to the wildtype (Figures 4A, 4B and data not shown). We also performed cortical patch to cytosol fluorescence intensity ratio analysis to assess whether there was any defect in localization of Pal proteins in these endocytic mutants. However, there was no significant difference in patch to cytosol fluorescence intensity ratio of the Pal proteins in the mutants compared to the wildtype strain (Figure S3A & S3B). These results suggest that the early endocytic factors tested are not required cortical recruitment factors for the Pal proteins. We also verified that Pal1 and Pal2 do not affect each other’s localization (≥78% of cells had cortical patches; n = 45, upon deletion of the paralog; Figure 4A & 4B), and thus their recruitment to the cell surface is not interdependent.

**Figure 5:**
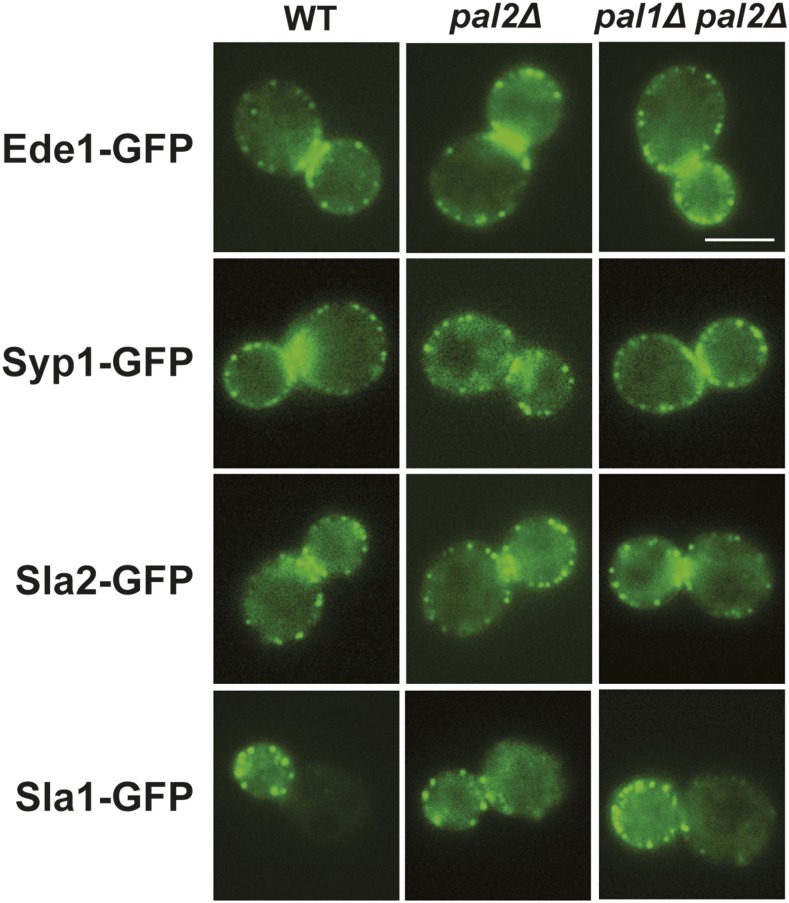
Recruitment of endocytic factors does not depend on Pal2 and Pal1. Localization of Ede1-GFP (WT; SL5755), Syp1-GFP (WT; SL5806), Sla2-GFP (WT; SL5927), Sla1-GFP (WT; SL5311); in *pal2∆* (SL 7330; 7298; 7295 and 7301 respectively) and in *pal1∆ pal2∆* (SL 7500; 7473; 7497 and 7476 respectively). Scale bar: 5μm.

To determine whether Pal2 and Pal1 have a role in the recruitment of other endocytic factors, we examined the localization of GFP tagged Ede1, Syp1, Sla2 and Sla1 in *pal2∆* or *pal1∆ pal2∆* cells. However, no significant difference in the localization of these endocytic factors was observed compared to wild type (Figure 5). We also noted that there was more cytosolic Sla1 in *pal2∆* or *pal1∆ pal2∆* cells (Figure 5 and see cortical patch to cytosol fluorescence intensity ratio in Figure S4). Altogether, our data suggest that Pal2 and Pal1 localization at endocytic sites is independent of early endocytic factors and both proteins are not required for recruitment of Ede1, Syp1 and Sla2, but they affect recruitment of Sla1 to the cortex during the immobile phase of internalization.

### Pal2 localizes to cortical patches containing other endocytic factors

**Figure 6:**
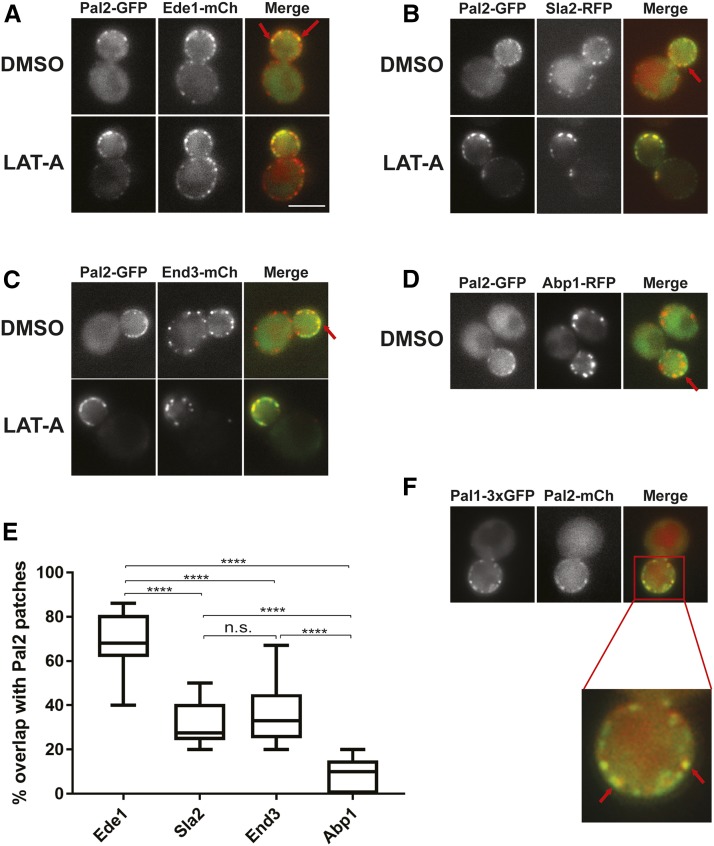
Localization of Pal2-GFP with other endocytic factors. Representative micrographs of yeast cells co-expressing Pal2-GFP with Ede1-mCherry (A; SL7483), Sla2-RFP (B; SL7479), End3-mCherry (C; SL7487), Abp1-RFP (D; SL7411) grown to log phase and treated for 1 h with DMSO vehicle control or 200 μM Latrunculin A (LAT-A). Red arrows indicate examples of co-localization. (E) Whisker plot showing the percentage overlap of Ede1, Sla2, End3 and Abp1 patches with Pal2 patches. Two-tailed *t*-test was performed for all the pairs. Except for the Sla2-End3 pair which is not significant (n.s.), all other pairs were significant at *P* < 0.0001 indicated by “****”. (F). Co-localization of Pal2-mCherry and Pal1-3xGFP (SL7459). Images shown are from a single medial plane of a z-axis collection. Scale bar: 5μm.

To determine whether Pal2 is actually in endocytic sites at the cortex, we tested for co-localization of Pal2-GFP with endocytic factors that mark different phases of clathrin-mediated endocytosis. These include Ede1 – early immobile phase, Sla2 and End3 – mid/late immobile phase, and Abp1 – actin/mobile phase (Boettner *et al.* 2011). To enhance any co-localization signal, we treated cells with Latrunculin-A (LAT-A), a drug that inhibits actin polymerization by sequestering monomers of actin and thereby stalling the endocytosis process (Ayscough 1998). The Pal2-GFP signal overlapped significantly with Ede1-mCherry, Sla2-mCherry and End3-mCherry, even in the absence of LAT-A (DMSO control cells) (Figure 6A – 6C). However, Pal2-GFP appeared to be largely at the surface more like Ede1 and not in invaginating structures. Consistent with this Pal2-GFP did not show significant overlap with Abp1-RFP (Figure 6D), which marks the mobile invagination phase of internalization and arrives at or just after Syp1 and Ede1 leave the cell surface (Boettner *et al.* 2009, Stimpson *et al.* 2009).

We also quantified the number of Pal2 patches that contained the early (Ede1) and middle/late endocytic coat factors (Sla2/End3), as well as actin (Abp1). Consistent with Pal2 arriving early at endocytic sites, 68% of Pal2 patches contained Ede1 (n = 111 from 18 cells), 31% contained Sla2 (n = 67 from 12 cells), 35% contained End3 (n = 56 from 13 cells), and only 8% contained Abp1 (n = 77 from 11 cells) (Figure 6E). As predicted, Pal1-3xGFP and Pal2 tagged with mCherry also colocalized at cortical sites (67%; n = 78 from 11 cells; Figure 6F). This supports the idea that Pal2 arrives before Sla2, End3 and Abp1. Overall these results suggest that Pal2 is an early endocytic factor that may leave the cortex without internalization, possibly similar to Ede1 and Syp1(Boettner *et al.* 2009, Reider *et al.* 2009, Stimpson *et al.* 2009).

### Eps15 homology (EH)-domain containing protein, Ede1, interacts with Pal2 and Pal1

**Figure 7:**
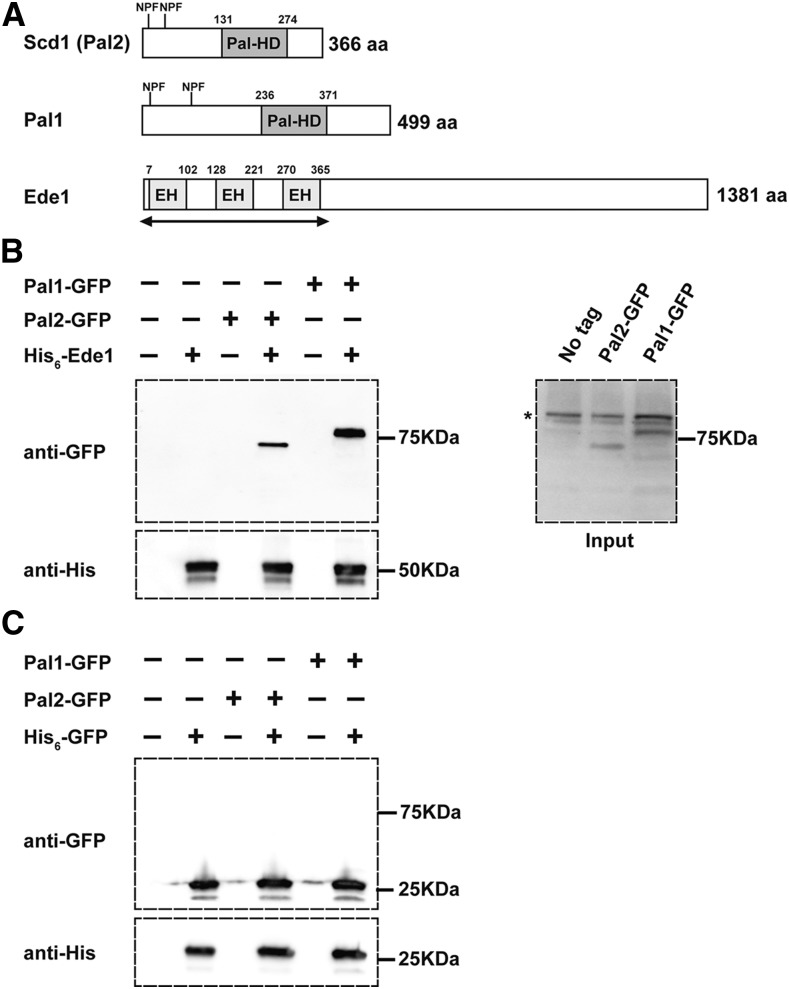
Interaction between Pal2-GFP and Pal1-GFP with His_6_-Ede1(EH1-3). (A) Schematic representation of Pal2, Pal1 and Ede1 proteins showing the Pal homology domain and NPF motifs in Pal2 and Pal1; and 3 EH-domains in Ede1. The double arrow under the Ede1 EH domain is the region used in the pulldown experiments in panel B. (B) Protein lysates from yeast cells expressing Pal2-GFP (SL7455) or Pal1-GFP (SL7442) were incubated with Ni-NTA agarose beads containing recombinant His_6_-Ede1(EH1-3). Both Pal2 and Pal1 co-purify with Ede1 and are detected by anti-GFP antibody. Inputs containing Pal2-GFP and Pal1-GFP are shown at the right; ‘*’ shows non-specific bands. (C) Protein lysates as in (B) from yeast cells expressing Pal2-GFP or Pal1-GFP were incubated with Ni-NTA agarose beads containing recombinant His_6_-GFP (His_6_-GFP) as a control. Neither Pal2 nor Pal1 co-purifies with His_6_-GFP.

Ede1, the homolog of mammalian Eps15 (Gagny *et al.* 2000), contains 3 N-terminal EPS15 homology (EH) domains (EH1: 7-102; EH2: 128-221; EH3: 270-365) which recognize the Asn-Pro-Phe (NPF) motifs (Figure 7A) in target ligands (de Beer *et al.* 2000, Gagny *et al.* 2000, Morgan *et al.* 2003, Suzuki *et al.* 2012). A previous study used immunoprecipitation to show Pal1 interacts with Ede1 in whole cell extracts (Carroll *et al.* 2012). To investigate whether Pal2 interacts with Ede1, we generated a recombinant protein expressing only the 3 EH domains (EH1-3) of Ede1 fused to a His_6_-tag (His_6_-Ede1 (EH1-3)) for pull downs of Pal1-1xGFP or Pal2-1xGFP from yeast cell extracts. Both Pal2 and Pal1 associated with His_6_-Ede1 (EH1-3) (Figure 7B), but not a His_6_-tagged-GFP control (Figure 7C). Our results indicate that both Pal1 and Pal2 associate with the N-terminal EH domain region of Ede1.

## Discussion

It has been over 30 years since the identification of a polymorphism in the gene referred to as *SCD1*, suppressor of clathrin deficiency, where one allele (*scd1-i*) resulted in inviability of clathrin HC deficient yeast, but the other (*scd1-v*) allowed survival of cells lacking clathrin HC (Lemmon and Jones 1987). At the time this was surprising because the concept of synthetic lethality in yeast was relatively novel and there were limited examples in the literature (Huffaker *et al.* 1987, Kaiser and Schekman 1990). It was also unanticipated to discover this random polymorphism in what were considered to be relatively isogenic yeast strains (Mortimer and Johnston 1986). Controversy over this finding ensued because it was unexpected that yeast cells should even survive without clathrin (Payne and Schekman 1985, Lemmon and Jones 1987, Payne *et al.* 1987, Schekman and Payne 1988, Munn *et al.* 1991, Robinson 2015). Indeed, viable *chc1∆* yeast exhibit very severe growth defects and a number of trafficking and other cell phenotypes, so their survival was considered tenuous at best. In fact, it was argued that mutations in many additional genes might cause a very sick strain to be inviable, and thus the possibility that the *SCD1* locus would have anything to do with clathrin function seemed remote (Payne *et al.* 1987, Schekman and Payne 1988, Munn *et al.* 1991). Moreover, use of the term “suppressor” for this polymorphism, though genetically valid, may have implied to some that the “mutant allele” was a viability conferring “suppressing” allele (*scd1-v*) (Lemmon and Jones 1987, Schekman and Payne 1988) (see response by Lemmon & Jones to Schekman & Payne 1988). Also, some studies showed that knockouts of *CHC1* in a number of different yeast strains usually led to viability of Chc-deficient yeast (Payne *et al.* 1987, Schekman and Payne 1988, Munn *et al.* 1991). This was used as an argument that *scd1-i* was the less common allele and likely an inactivating mutation in *SCD1*. However, a number of mutations are propagated in our commonly used yeast strains to make them more amenable to genetic manipulation, so this still left the nature of the *SCD1* locus and the *scd1-i* allele unclear (see response of Lemmon & Jones to (Schekman and Payne 1988)).

The search for the *SCD1* locus proved to be highly challenging. We initially used a multicopy suppression strategy, seeking genes whose overexpression could suppress the lethality of *scd1-i* clathrin HC-deficient cells in which *CHC1* was under control of the repressible *GAL1* promoter (Nelson and Lemmon 1993). We reasoned that overexpression might overcome the need to know which allele of *SCD1* was dominant or recessive. In the process we identified *SCD2-SCD6* and uncovered very interesting biology, including genes encoding proteins that linked directly to membrane trafficking and endocytosis (Nelson and Lemmon 1993, Gelperin *et al.* 1995, Nelson *et al.* 1996, Huang *et al.* 1997, Chang *et al.* 2002, Henry *et al.* 2002, Henry *et al.* 2003, Newpher *et al.* 2006, Chi *et al.* 2012). However, genetic analysis demonstrated that none of these multicopy suppressors are allelic to the *SCD1* locus ((Nelson and Lemmon 1993, Gelperin *et al.* 1995, Nelson *et al.* 1996, Huang *et al.* 1997); Gelperin and S. Lemmon unpublished). A caveat to this approach was we did not know the nature of the *SCD1* locus in existing plasmid libraries. In addition, other genetic mapping strategies were thwarted by the propensity of clathrin-deficient yeast to become polyploid and their poor transformation efficiency (Lemmon and Jones 1987, Lemmon *et al.* 1990).

The advent of powerful pooled linkage analysis and whole genome sequencing (Birkeland *et al.* 2010, Song *et al.* 2014, Lang *et al.* 2015, Linder *et al.* 2016) allowed us to finally identify the *SCD1* locus, solving this long unresolved question. We found that *scd1-i* is a mutation in *YHR097c*, also referred to as *PAL2* due to the existence of a paralogue, *PAL1* (Carroll *et al.* 2012, Brach *et al.* 2014). The *scd1-i* allele has a stop codon that results in a truncated protein, and the *scd1-v* allele is the wild type *SCD1*/*PAL2* gene. This confirms that it is a loss of function allele that is synthetic lethal with clathrin HC deficiency. Of particular interest, though, our data indicate that the Pal2/Scd1 protein plays a role in clathrin-mediated endocytosis, arguing against the concept that this mutation was not likely relevant to clathrin.

The first Pal1 protein was characterized in *S. pombe* cells, where it localizes to the cell tips during interphase and at the cell division plane during mitosis and cytokinesis (Ge *et al.* 2005). A null mutation causes morphological and polarity phenotypes, including pear shaped and spherical cells, thus the name Pal1 for **p**ears **a**nd **l**emons. A possible role in endocytosis was suggested by an association with Sla2, related to HIP1/R in mammalian cells (Ge *et al.* 2005). More recently Pal1 was studied in *S. cerevisiae* where it was found to localize to sites of clathrin-mediated endocytosis and behave like an early endocytic coat factor (Carroll *et al.* 2012). Our studies here indicate its paralog, Pal2, also may play an endocytic role. It localizes to cortical patches containing other endocytic coat factors, including Pal1, and it interacts with the early coat factor Ede1, like Pal1. For technical reasons, we have not been able to study Pal2’s dynamics by live cell imaging. However, it appears to localize more like early endocytic factors that do not internalize, such as Ede1 and Syp1. There was no obvious displacement from the cortex, and limited colocalization with actin as seen in factors found in invaginating vesicles. This would be a distinction from Pal1 which seems to internalize with endocytic vesicles (Carroll *et al.* 2012).

A few questions still need to be answered about the Pal proteins in yeast. First, are Pal1 and Pal2 redundant? Arguing against this idea is *pal2∆* is synthetic lethal with *chc1∆*, while *pal1∆* is not. However, it could be that Pal2 is just more abundant than Pal1, so depletion of Pal2 has more of an effect in the absence of clathrin HC. However, proteomic quantification of the number of Pal proteins per cell showed little difference in abundance (Saccharomyces Genome Database, (Kulak *et al.* 2014)).

Furthermore, we still don’t know what are the exact function(s) of the Pal proteins. There is no obvious phenotype of the null mutations alone or in combination, or combined with other early endocytic factors tested so far, except for the genetic interaction of *PAL2* with *CHC1*. Although, further supporting an endocytic role for the Pal proteins, *pal2∆* and *pal1∆pal2∆* did lead to an increase in the cytoplasmic level of Sla1 compared to the wild type strain. This is similar to the effects seen on Sla1 when genes for other early endocytic factors are deleted (Kaksonen *et al.* 2005, Brach *et al.* 2014). Also, both proteins have NPF motifs and bind to the EH region of Ede1. Prior studies suggested that Ede1 is needed to recruit Pal1 to the cell cortex (Carroll *et al.* 2012), but our results did not confirm these findings, as both Pal1 and Pal2 were in cortical patches without Ede1.

It is possible that the Pal proteins have an endocytic function that is only critical under certain conditions, such as cell stress or during morphogenesis events like mating. Alternatively, they are selective cargo adaptors for membrane proteins that have yet to be identified. Deletions of AP1801/2, yeast AP2, and Syp1 adaptors cause no major general growth and limited endocytic defects (Huang *et al.* 1999, Kaksonen *et al.* 2005, Boettner *et al.* 2009, Stimpson *et al.* 2009), but they participate in internalization of specific cargos. AP180’s are adaptors for Snc1, a vesicle SNARE (Burston *et al.* 2009), while AP2 is important for Killer Toxin toxicity (Carroll *et al.* 2009) and for internalization of the stress sensor Mid2 (Chapa-y-Lazo *et al.* 2014). Mid2 is also a cargo of Syp1 (Reider *et al.* 2009), a F-Bar -µHomology domain protein. Syp1’s uHomology region was recently shown to interact with cargo with DxY motifs, including those in Mid2, Snc1, Ptr2 and Mep3 (Apel *et al.* 2017). Perhaps the Pal1 homology domain also binds specific cargo motifs. Further studies will be needed to investigate these possibilities and to understand the roles of the Pal proteins.
